# Blood pressure control in chronic kidney disease: A cross-sectional analysis from the German Chronic Kidney Disease (GCKD) study

**DOI:** 10.1371/journal.pone.0202604

**Published:** 2018-08-20

**Authors:** Markus P. Schneider, Karl F. Hilgers, Matthias Schmid, Silvia Hübner, Jennifer Nadal, David Seitz, Martin Busch, Hermann Haller, Anna Köttgen, Florian Kronenberg, Seema Baid-Agrawal, Georg Schlieper, Ulla Schultheiss, Thomas Sitter, Claudia Sommerer, Stephanie Titze, Heike Meiselbach, Christoph Wanner, Kai-Uwe Eckardt, for the GCKD Study Investigators

**Affiliations:** 1 Department of Nephrology and Hypertension, University Hospital Erlangen, Friedrich-Alexander Universität Erlangen-Nürnberg, Erlangen, Germany; 2 Department of Nephrology and Hypertension, Klinikum Nürnberg, Paracelsus Private Medical University, Nürnberg, Germany; 3 Department of Medical Biometry, Informatics, and Epidemiology (IMBIE), University of Bonn, Bonn, Germany; 4 Department of Internal Medicine III, University Hospital Jena, Friedrich-Schiller-Universität, Jena, Germany; 5 Division of Nephrology, Hannover Medical School, Hannover, Germany; 6 Division of Genetic Epidemiology, Institute for Biometry and Statistics, Faculty of Medicine and Medical Center—University of Freiburg, Freiburg, Germany; 7 Division of Genetic Epidemiology, Department of Medical Genetics, Molecular and Clinical Pharmacology, Innsbruck Medical University, Innsbruck, Austria; 8 Department of Nephrology and Medical Intensive Care, Charité –Universitätsmedizin Berlin, Berlin, Germany; 9 Department of Nephrology and Clinical Immunology, RWTH Aachen, Aachen, Germany; 10 Division of Nephrology, University of Freiburg, Faculty of Medicine and Medical Center—University of Freiburg, Freiburg, Germany; 11 Department of Nephrology, University Hospital, Ludwig-Maximilians-Universität München, München, Germany; 12 Department of Nephrology, University of Heidelberg, Heidelberg, Germany; 13 Division of Nephrology, Department of Medicine, University Hospital of Würzburg, Würzburg, Germany; International University of Health and Welfare, School of Medicine, JAPAN

## Abstract

We assessed the prevalence, awareness, treatment and control of hypertension in patients with moderate chronic kidney disease (CKD) under nephrological care in Germany. In the German Chronic Kidney Disease (GCKD) study, 5217 patients under nephrology specialist care were enrolled from 2010 to 2012 in a prospective observational cohort study. Inclusion criteria were an estimated glomerular filtration rate (eGFR) of 30–60 mL/min/1.73 m^2^ or overt proteinuria in the presence of an eGFR>60 mL/min/1.73 m^2^. Office blood pressure was measured by trained study personnel in a standardized way and hypertension awareness and medication were assessed during standardized interviews. Blood pressure was considered as controlled if systolic < 140 and diastolic < 90 mmHg. In 5183 patients in whom measurements were available, mean blood pressure was 139.5 ± 20.4 / 79.3 ± 11.8 mmHg; 4985 (96.2%) of the patients were hypertensive. Awareness and treatment rates were > 90%. However, only 2456 (49.3%) of the hypertensive patients had controlled blood pressure. About half (51.0%) of the patients with uncontrolled blood pressure met criteria for resistant hypertension. Factors associated with better odds for controlled blood pressure in multivariate analyses included younger age, female sex, higher income, low or absent proteinuria, and use of certain classes of antihypertensive medication. We conclude that blood pressure control of CKD patients remains challenging even in the setting of nephrology specialist care, despite high rates of awareness and medication use.

## Introduction

Chronic kidney disease (CKD) has been increasingly recognized as a global health burden [1, 2]. The prevalence of CKD is 10–15% in the general adult population in both high- and low-income countries. Individuals with CKD are at risk for progressive loss of kidney function and kidney failure requiring renal replacement therapy. In addition, the presence of CKD worsens the prognosis of many non-renal diseases. In particular, CKD has been recognized as a major cardiovascular risk factor [3].

Blood pressure control is considered a mainstay of CKD management, in order to alleviate the progressive loss of kidney function, as well as to lower the risk of cardiovascular disease [4]. The controversial discussion about optimal blood pressure targets in CKD patients [5, 6] has recently been rekindled by the results of the SPRINT trial in which 28% of enrolled patients had CKD [7]. Several authors now recommend to lower blood pressure goals in patients with CKD [8, 9]. All recent guidelines developed prior to publication of the SPRINT trial propose maintaining blood pressure at least below 140/90 mmHg in all CKD patients [10–12]. For patients with CKD and proteinuria above 300 mg/L, most guidelines [10, 11] suggest lower blood pressure targets, although the evidence supporting this suggestion is limited [5, 6]. Despite these recommendations, studies from large population-based surveys and CKD cohorts in the United States [13, 14] as well as from western European countries [15, 16] have shown rather poor rates of blood pressure control.

We enrolled >5200 patients treated by nephrologists into the German Chronic Kidney Disease (GCKD) study, a long-term observational cohort study [17]. A cross-sectional overview of patient characteristics at baseline [18] showed rather high blood pressure levels, despite the fact that >80% of patients had been under nephrology specialist care for at least 1 year. To better understand the limitations of blood pressure control in this population we have therefore analyzed associations with comorbidities, sociodemographic factors, medication patterns and the presence of resistant hypertension. Our results describe the diversity of blood pressure control and overall emphasize the need to alleviate the burden of hypertension in CKD patients.

## Materials and methods

### Study participants

The design of the study has been previously published in detail [17]. The GCKD study was registered in a national database of clinical studies (“Deutsches Register für Klinische Studien”, DRKS 00003971), and was approved by the ethics committees of all participating centers (Friedrich Alexander University of Erlangen-Nuremberg, University of Freiburg, Ludwig-MaximiliansUniversity of Munich, University of Hannover, Charité –Universitätsmedizin Berlin, University of Würzburg, University of Aachen, University of Jena, Heidelberg University). Caucasian patients could be included into the study if age was 18 to 74 years, and if they met at least one of two criteria: a) an estimated glomerular filtration rate (eGFR) between 30 and 60 ml/min/1.73 m^2^ or b) manifest proteinuria (urinary protein/creatinine >500 mg/g or equivalent) if eGFR was >60 ml/min/1.73 m^2^. Patients were not able to participate if they previously underwent solid organ or bone marrow transplantation, had suffered a malignancy, had advanced heart failure, were under legal attendance or not able to provide informed consent. Written informed consent was obtained from all participants prior to their enrolment.

### Examinations performed

The patients were examined by certified study nurses in the regional GCKD centers or in the nephrology practices of the participating nephrologists. The training of study nurses included the correct and standardized conduct of blood pressure measurements. Prior to the blood pressure measurements, upper arm circumference was determined in order to use the correct cuff size (small, medium, large and extra-large cuffs were available). Blood pressure was measured in a sitting position after 5 min of rest with an oscillometric device (Omron M5 Professional devices). Three measurements were taken in the presence of the study nurse (“observed measurements”) with two minutes between each measurement. The mean of the three measurements went into the current analysis. No specific instructions were given with regard to caffeine or tobacco intake on the day of the study visit. In addition, detailed information on the medical history and sociodemographic factors was obtained. Definitions of co-morbidities etc. were used according to international standards [18]. Information on the precise type of any medication (prescribed and over the counter) was collected, but dosage and intake schedules were not recorded. Substances were classified using the WHO’s Anatomical Therapeutic Chemical (ATC) classification (http://www.whocc.no/). For the purpose of our analysis, all antihypertensive medications were grouped into the following 7 main categories: (1) inhibitors of the renin-angiotensin system (RAS; comprising the 3 sub-categories ACE inhibitors, AT1 blockers, and renin inhibitors); (2) diuretics (comprising the 3 sub-categories thiazide diuretics, loop diuretics, and potassium-sparing diuretics including aldosterone blockers), (3) beta blockers, (4) central sympatholytic agents, (5) peripheral acting alpha-adrenergic antagonists, (6) calcium channel blockers, and (7) other vasodilators. All data presented in this manuscript (including all blood pressure data and all covariates) were taken from the GCKD baseline visit.

### Definitions of hypertension, awareness, control, and resistant hypertension

Patients were considered “**hypertensive**” if the mean of the 3 blood pressure measurements was ≥ 140 mmHg systolic, or ≥ 90 mmHg diastolic, or if any antihypertensive drug was currently prescribed. A hypertensive patient was defined as “**aware**” of hypertension if he or she described him- or herself as hypertensive in the patient questionnaire during the interview. The blood pressure was defined as “**controlled**” if the mean of 3 recordings yielded < 140 mmHg systolic and < 90 mmHg diastolic in a patient classified as hypertensive. A hypertensive patient was considered to suffer from “**resistant hypertension**” if the mean of the 3 blood pressure measurements was ≥ 140 mmHg systolic, or ≥ 90 mmHg diastolic, despite a current medication with at least 3 different antihypertensive drugs, including a diuretic.

### Statistical analysis

Data is described using means ± standard deviations (SD) for continuous variables and frequency distributions with percentages for categorical variables. Odds ratios with 95% confidence intervals were calculated to analyze the univariate associations (or lack thereof) of controlled blood pressure with a number of factors, including antihypertensive medications (i.e., the groups described above), age category (≥18- <45,≥ 45- < 65 and ≥65 years old), sex, marital status (single, married, divorced/separated, widowed), education, gross yearly income, type of health insurance (statutory or private), body mass index (underweight: BMI < 18.5; normal weight: BMI ≥18.5 to <25; overweight: BMI ≥25 to <30; obesity: BMI≥30), smoking status (non-smoker, former smoker, current smoker), self-reported ethanol intake, diabetes mellitus (absence or presence), previous cardiovascular, cerebrovascular or peripheral vascular disease, stratum of urine albumin-to-creatinine ratio (< 30 mg/g, ≥30- <300 mg/g, ≥ 300 mg/g), and eGFR stratum (≥60, ≥45-< 60, ≥30- <45, <30 ml/min/1.73m^2^) estimated according to the 4-variable Modification of Diet in Renal Disease formula [19]. For multivariate analysis, adjusted odds ratios with 95% confidence intervals for all factors were calculated using the coefficient estimates obtained from binary logistic regression. A p-value < 0.05 was considered significant. Calculations were carried out using SAS Version 9.2 by SAS Institute Inc., Cary NC, USA.

## Results

**Fig 1:**
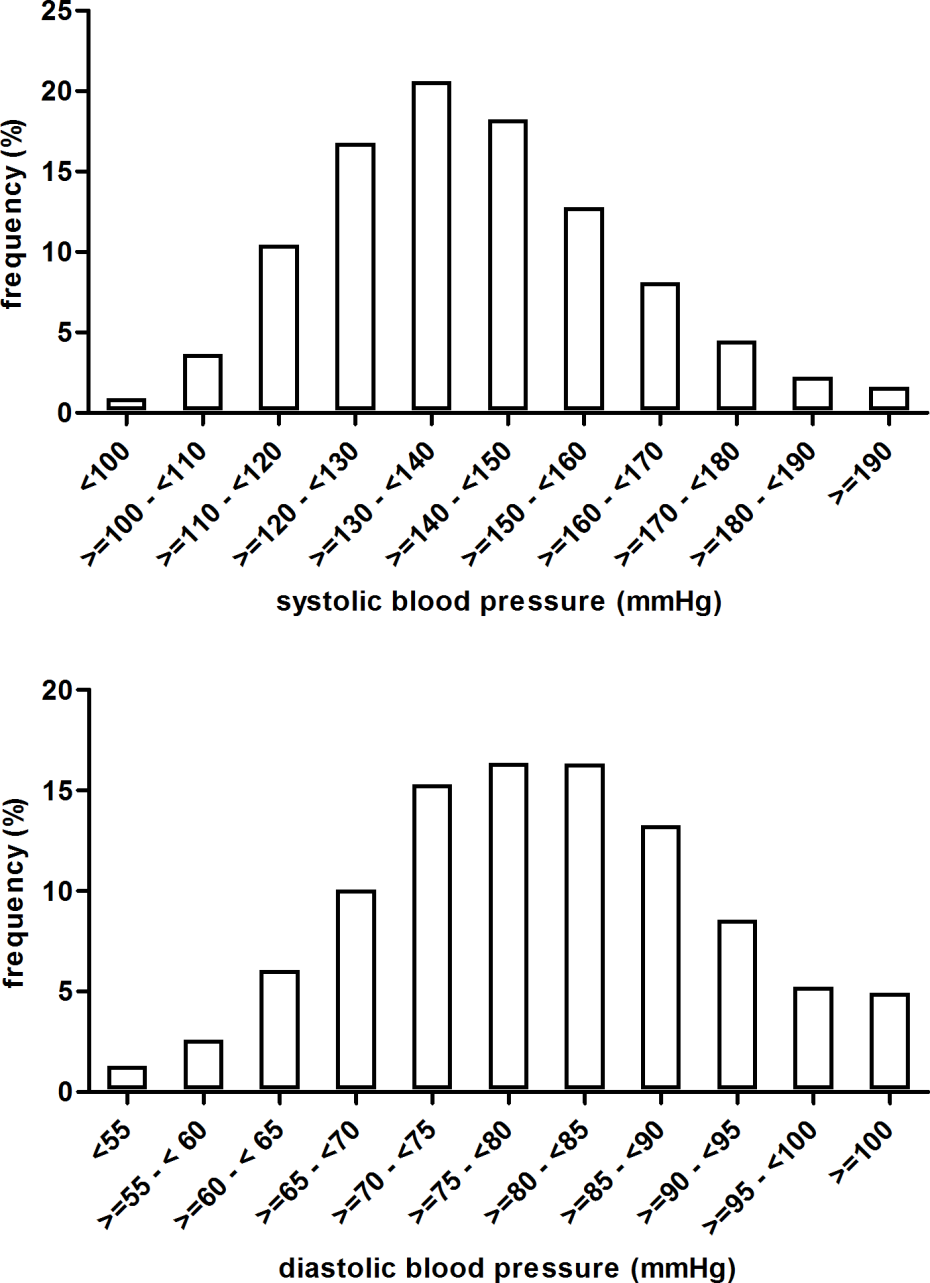
Frequency distribution of systolic (upper panel) and diastolic (lower panel) blood pressure in participants of the GCKD study.

Blood pressure data were available for analysis from 5183 participants. Mean ± SD blood pressure was 139.5 ± 20.4 / 79.3 ± 11.8 mmHg (N = 5183). The distribution of blood pressure levels in the entire cohort is shown in Fig 1. Mean blood pressure levels for subgroups defined by age, sex, eGFR stratum, proteinuria stratum and presence of diabetes are shown in Table 1. No fewer than 4985 of 5183 (96.2%) met the criteria for hypertension.

**Table 1:** Blood pressure levels and control of blood pressure according to demographic, biometric and other factors.

	Patients number (%)	SBP (mmHg)	DBP (mmHg)	Patients with Hypertension (%)	Uncontrolled (%)	Controlled (%)
**Entire cohort**	5183 (100)	139.5 ± 20.4	79.3 ± 11.8	4985 (96.2)	2529 (48.8)	2456 (47.4)
**Age (years)**						
≥18 - < 45	632 (12.2)	129.6 ± 15.2	81.3 ± 11.5	579 (11.2)	193 (3.7)	386 (7.4)
≥ 45 - < 65	2134 (41.2)	138.1 ± 19.5	81.9 ± 11.5	2039 (39.3)	1008 (19.4)	1031 (19.9)
≥ 65	2417 (46.6)	143.3 ± 21.2	76.4 ± 11.4	2367 (45.7)	1328 (25.6)	1039 (20.0)
**Sex**						
Male	3115 (60.1)	141.7 ± 20.1	79.3 ± 11.9	3050 (58.8)	1662 (32.1)	1388 (26.8)
Female	2068 (39.9)	136.2 ± 20.3	79.2 ± 11.5	1935 (37.3)	867 (16.7)	1068 (20.6)
**Diabetes mellitus**						
No	3345 (64.5)	138.1 ± 19.7	80.9 ± 11.4	3165 (61.1)	1544 (26.8)	835 (16.1)
Yes	1838 (35.5)	142.0 ± 21.3	76.3 ± 11.8	1820 (35.1)	985 (19.0)	1621 (31.3)
**eGFR (ml/min/1.73m**^**2**^**) **						
≥ 60	1115 (21.8)	136.4 ± 19.4	80.8 ± 10.9	1031 (20.1)	467 (9.1)	564 (11.0)
≥ 45 - < 60	1708 (33.3)	140.4 ± 20.1	80.0 ± 11.5	1633 (31.9)	870 (17.0)	763 (14.9)
≥ 30 - < 45	1846 (36.0)	140.3 ± 20.6	78.4 ± 12.0	1815 (35.4)	929 (18.1)	886 (17.3)
< 30	458 (8.9)	139.8 ± 21.9	76.5 ± 12.9	455 (8.9)	230 (4.5)	225 (4.4)
**Albuminuria (mg/g)**						
< 30	2176 (42.7)	136.5 ± 19.2	77.2 ± 11.4	2046 (40.2)	932 (18.3)	1114 (21.9)
≥ 30 - < 300	1485 (29.2)	140.3 ± 20.3	79.5 ± 11.9	1442 (28.3)	747 (14.7)	695 (23.6)
≥ 300	1434 (28.1)	143.2 ± 21.4	82.2 ± 11.4	1412 (27.7)	804 (15.8)	608 (11.9)
**Marital Status**						
Single	478 (9.2)	137.1 ± 20.4	81.1 ± 11.7	456 (8.8)	203 (3.9)	253 (4.9)
Married	3991 (77.0)	139.7 ± 20.0	79.1 ± 11.7	3841 (74.1)	1978 (38.2)	1863 (35.9)
Separated/ divorced	370 (7.1)	137.7 ± 22.0	80.6 ± 12.1	349 (6.7)	168 (3.2)	181 (3.5)
Widowed	344 (6.6)	142.4 ± 22.3	77.3 ± 12.2	339 (6.5)	180 (3.5)	159 (3.1)
**Education Level**						
Low	2765 (53.4)	141.1 ± 20.9	78.4 ± 11.7	2699 (52.1)	1429 (27.6)	1270 (24.5)
Intermediate	1446 (27.9)	137.9 ± 19.7	80.2 ± 11.6	1364 (26.3)	660 (12.7)	704 (13.6)
High	868 (16.8)	137.3 ± 19.4	80.4 ± 12.0	821 (15.8)	392 (7.6)	429 (8.3)
other	104 (2.0)	138.0 ± 19.7	79.2 ± 11.3	101 (1.9)	48 (0.9)	53 (1.0)
**Annual gross income**						
< 25.000 €	1766 (46.7)	141.6 ± 20.7	78.3 ± 11.8	1720 (45.4)	939 (24.8)	781 (20.6)
25.000–50.000€	1404 (37.1)	138.0 ± 19.1	79.3 ± 11.2	1344 (35.5)	645 (17.0)	699 (18.5)
50.000–100.000€	528 (14.0)	136.5 ± 19.4	81.4 ± 11.7	497 (13.1)	232 (6.1)	265 (7.0)
> 100.00€	87 (2.3)	133.0 ± 16.2	80.7 ± 11.4	76 (2.0)	32 (0.8)	44 (1.2)
**Insurance status**						
Statutory	4822 (93.0)	139.6 ± 20.3	79.2 ± 11.7	4639 (89.5)	2359 (45.5)	2280 (44.0)
Private	358 (6.9)	138.5 ± 20.3	80.1 ± 11.8	343 (6.6)	168 (3.2)	175 (3.4)
unkown	3 (0.1)	134.7 ± 19.9	83.0 ± 11.0	3 (0.1)	2 (0.1)	1 (0.1)
**Body Mass Index**						
<18.5	32 (0.6)	124.1 ± 21.5	75.2 ± 14.1	24 (0.5)	6 (0.1)	18 (0.4)
≥18.5 - <25	1012 (19.7)	136.0 ± 19.7	79.3 ± 11.1	927 (18.1)	421 (8.2)	506 (9.9)
≥25 - <30	1882 (36.7)	140.7 ± 20.3	79.8 ± 11.5	1816 (35.4)	960 (18.7)	856 (16.7)
≥30	2206 (43.0)	140.2 ± 20.4	78.8 ± 12.2	2168 (42.2)	1113 (21.7)	1055 (20.6)
**Smoking status**						
Non-smoker	2116 (40.9)	139.3 ± 19.6	79.8 ± 11.6	2023 (39.1)	1022 (19.8)	1001 (19.4)
Former smoker	2232 (43.2)	140.5 ± 21.1	78.7 ± 11.8	2161 (41.8)	359 (6.9)	429 (8.3)
Current smoker	822 (15.9)	137.1 ± 20.2	79.2 ± 12.0	788 (15.2)	1141 (22.1)	1020 (19.7)
**Alcohol intake**						
No or very little	4173 (81.0)	138.8 ± 20.5	79.6 ± 11.8	4006 (77.7)	1982 (38.4)	2024 (39.3)
Moderate or high	982 (19.0)	142.1 ± 19.4	79.7 ± 11.5	951 (18.4)	529 (10.3)	422 (8.2)
**Cardiovascular disease**						
No	3517 (67.9)	138.8 ± 19.9	80.7 ± 11.5	3341 (64.5)	1677 (32.4)	1664 (32.1)
Yes	1666 (32.1)	140.9 ± 21.2	76.2 ± 11.7	1644 (31.7)	852 (31.7)	792 (31.7)
**Cerebrovascular disease**						
No	4680 (90.3)	139.3 ± 20.2	79.6 ± 11.7	4488 (86.6)	2264 (43.7)	2224 (42.9)
Yes	503 (9.7)	141.7 ± 21.5	76.6 ± 12.3	497 (9.6)	265 (5.1)	232 (4.5)
**Peripheral vascular disease**						
No	4695 (90.6)	139.1 ± 20.3	79.7 ± 11.7	4507 (87.0)	2255 (43.5)	2252 (43.4)
Yes	488 (9.4)	143.2 ± 20.8	75.4 ± 11.3	478 (9.2)	274 (5.3)	204 (3.9)

The frequency of the respective strata for the analyzed variables is indicated for all participants, participants with hypertension, hypertensive participants with uncontrolled hypertension (≥140/90 mmHg), and participants with controlled hypertension (<140/90 mmHg). The percentage numbers (in brackets) indicate percent of all participants in which the respective variable was known. SBP = Systolic blood pressure, DBP = Diastolic blood pressure.

In the univariate analysis, several factors were associated with the prevalence of hypertension but effects were rather small. For example, the prevalence rose with age from 91.6% in the 18–44 years group to 95.6% in the 45–64 years group and to 97.9% in the 65–74 years group. Effects of similar size were observed for gender (93.6% in women vs. 97.9% in men), education, income, eGFR and proteinuria stratum (higher prevalence in higher strata), body mass index, presence of diabetes (98.0% vs. 93.6% in non-diabetic patients) or history of cardiovascular disease. Remarkably, all subgroups in all analyses exhibited a prevalence of hypertension > 90% (except for two very small subgroups with < 100 participants: patients with gross yearly income in excess of 100.000 €, and patients with underweight).

**Fig 2:**
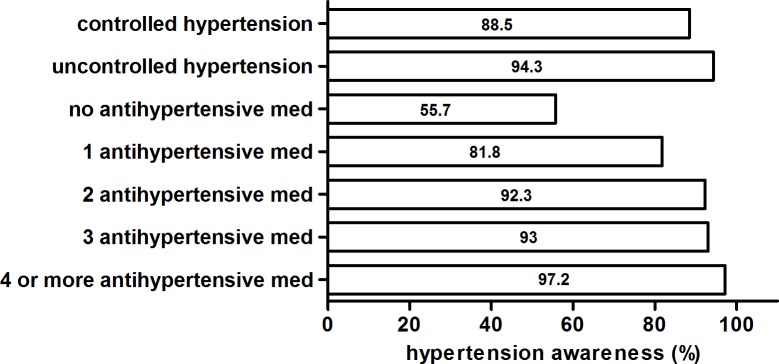
Awareness of hypertension according to control of hypertension and medication pattern.

Disease awareness was high among hypertensive patients (4557 of 4985 hypertensive participants, i.e. 94.1%). Fig 2 shows the awareness of hypertension according to control of hypertension and medication pattern. Patients with uncontrolled hypertension, and patients receiving multiple antihypertensive medications, had the highest rates of awareness. In contrast, hitherto untreated hypertensive patients (N = 127) with blood pressure levels ≥ 140 mmHg systolic or ≥ 90 mmHg diastolic exhibited low awareness.

Table 2 summarizes the utilization of different classes of antihypertensive medications. The vast majority (97.4%, N = 4854) of the hypertensive patients received at least one antihypertensive drug; only 127 patients had a blood pressure > 140/90 mmHg, but were not treated with antihypertensive medication. Most hypertensive patients (80.4%, N = 4008) received some form of combination therapy: Two (22.3%, N = 1111), three (23.3%, N = 1159), or more than three (34.9%, N = 1738) different antihypertensive drugs.

**Table 2:** Use of antihypertensive medications.

	Patients with hypertension (%)	Uncontrolled	Controlled
**Antihypertensive Medication**			
Yes	4858 (97.4)	2402 (95.0)	2456 (100.0)
No	127 (2.6)	127 (5.0)	0 (0.0)
**Antihypertensive medication sorted by substance class**			
**RAS inhibitors (total)**	4134 (82.9)	2026 (80.1)	2108 (85.9)
ACE inhibitors	2439 (48.9)	1140 (45.1)	1299 (52.9)
Angiotensin receptor blocker	2011 (40.3)	1031 (40.8)	980 (39.9)
Renin inhibitors	285 (5.7)	176 (6.9)	109 (4.4)
**Combined RAS blockade (total)**	585 (11.7)	313 (12.4)	272 (11.1)
ACEI and ARB	396 (7.9)	197 (7.8)	199 (8.1)
ACEI and renin inhibitor	84 (1.7)	48 (1.9)	36 (1.5)
ARB and renin inhibitor	105 (2.1)	68 (2.7)	37 (1.5)
ACEI, ARB and renin inhibitor	16 (0.3)	8 (0.3)	8 (0.3)
**Beta blocker**	2844 (57.1)	1474 (58.3)	1370 (55.8)
**Vasodilator**	219 (4.4)	114 (4.5)	105 (4.3)
**Ca channel blocker**	1951 (39.1)	1088 (43.0)	863 (35.1)
**Central Antiadrenergic**	627 (12.6)	403 (15.9)	224 (9.1)
**Peripheral Antiadrenergic**	327 (6.6)	215 (8.5)	112 (4.6)
**Diuretics (total)**	3119 (62.6)	1546 (61.1)	1573 (64.1)
Loop diuretic	1988 (39.9)	966 (38.2)	1022 (41.6)
Thiazides and analogs	1564 (31.4)	803 (31.8)	761 (30.1)
Potassium-sparing diuretics	501 (10.1)	200 (7.9)	301 (12.3)

Usage of a given drug class in percent was calculated in relation to all hypertensive patients, all hypertensive patients with uncontrolled blood pressure (≥140/90 mmHg), and all hypertensive patients with controlled blood pressure (<140/90 mmHg), respectively. RAS, renin-angiotensin system. ACEI, angiotensin converting enzyme inhibitors. ARB, angiotensin receptor blockers. RI, renin inhibitor. Ca, Calcium.

**Fig 3:**
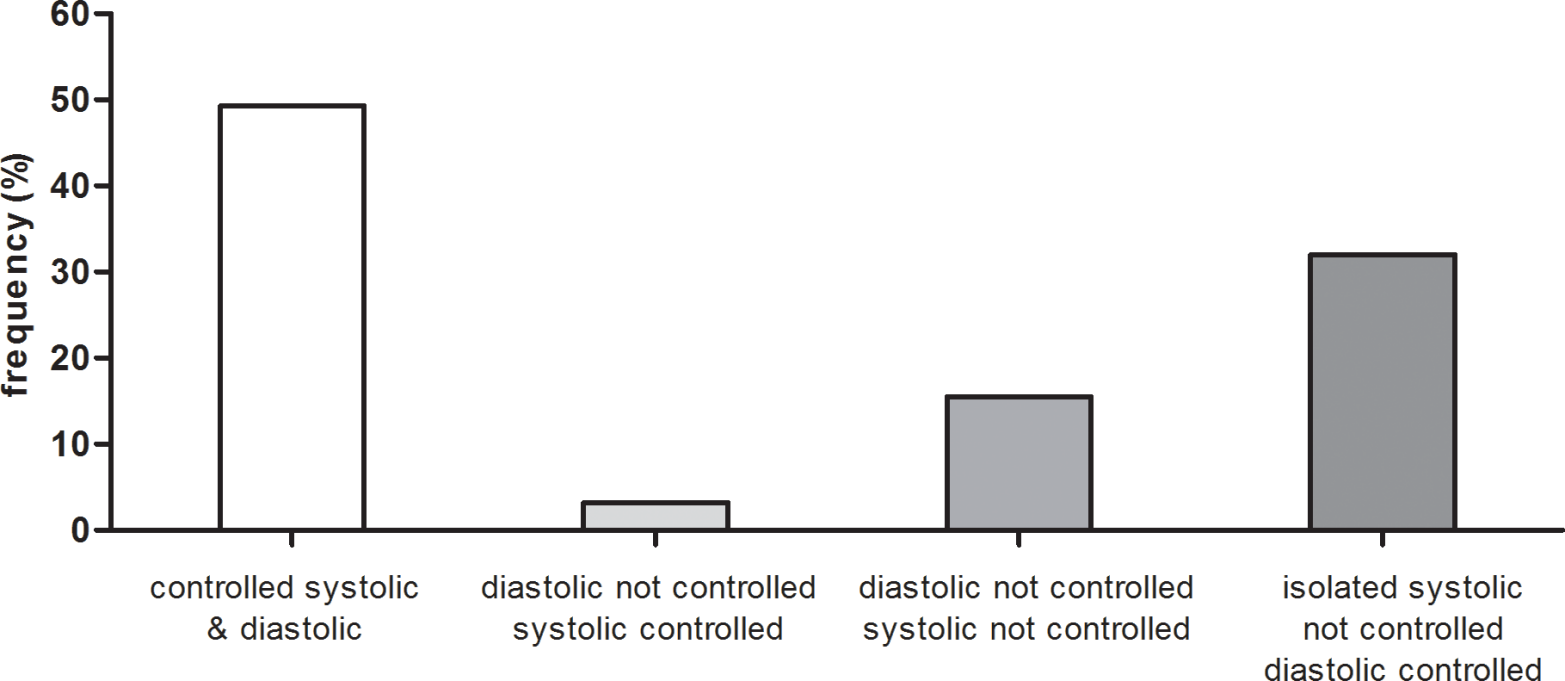
Control of blood pressure in hypertensive participants of the GCKD study.

**Fig 4:**
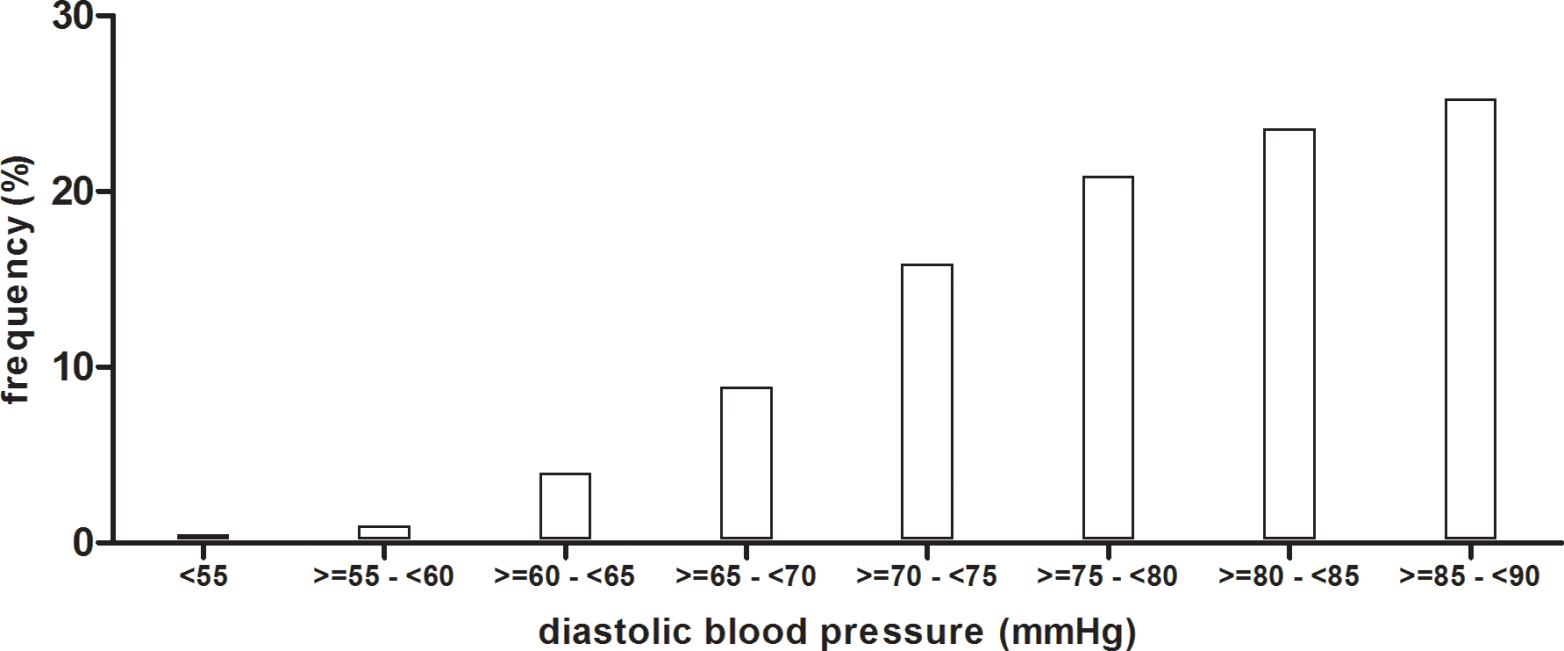
Frequency distribution of diastolic blood pressure levels of the 1596 patients with uncontrolled systolic but controlled diastolic blood pressure.

The control of hypertension, however, was rather low, despite the high awareness and treatment rates and the intense antihypertensive pharmacotherapy. Using the rather conservative blood pressure target level of < 140 mmHg systolic and < 90 mmHg diastolic, no fewer than 2529 of 4985 hypertensive patients (50.7%) failed to reach target levels. In most cases, the systolic component of blood pressure was not controlled (Fig 3). Reducing systolic blood pressure can be limited by the risk of a too low diastolic blood pressure. To assess the prevalence of very low diastolic pressure levels in this group, we plotted the diastolic blood pressure levels of the 1596 patients with uncontrolled systolic but controlled diastolic blood pressure (Fig 4). However, only 230 patients in this group had a diastolic blood pressure < 70 mmHg, suggesting that diastolic blood pressure level was not a main hurdle for intensification of blood pressure lowering in most patients with isolated systolic hypertension. In fact, roughly half of them had a diastolic blood pressure between 80 and 90 mmHg and a low diastolic blood pressure would not have been a hindrance for further blood pressure lowering.

In the univariate analysis (Table 3), several factors were associated with a lower odds ratio for successful control of blood pressure: higher age, male sex, lower education, lower income, higher BMI, higher ethanol intake, presence of diabetes mellitus or peripheral vascular disease, higher strata of proteinuria and lower eGFR. In the multivariate analysis (Table 3), some factors remained significant, exhibiting clearly lower odds for control of blood pressure: higher age (OR 0.532 (0.411–0.690) for age ≥45≤65 versus ≥18≤45 and OR 0.379 (0.285–0.505) for age ≥65 versus ≥18≤45, *P*<0.0001), male sex (OR 1.424 (1.208–1.678), *P*<0.0001), lower income (OR 1.273 (1.082–1.498) for annual gross income 25.000–50.000 € versus <25.000 €, OR 1.286 (1.011–1.636) for 50.000–100.000 € versus <25.000 €, and OR 1.635 (0.968–2.762) for >100.000 € versus <25.000 €, *P* = 0.0134), and higher proteinuria (OR 0.735 (0.621–0.871) for albuminuria ≥30≤300 versus <30 mg/g and OR 0.488 (0.406–0.587) for ≥300 versus <30 mg/g, *P*<0.0001). In contrast, eGFR strata, education, BMI, ethanol intake and presence of diabetes mellitus were no longer associated with blood pressure control in the multivariate analysis.

**Table 3:** Association of blood pressure control with demographic, biometric, pharmacologic and other factors.

	Univariate analysis		Multivariate analysis	
Variable	OR (95% CI)	p-value	OR (95% CI)	p-value
**Age**				
≥ 18 - < 45	Reference	< .0001	Reference	< .0001
≥ 45 - < 65	0.511 (0.422–0.621)		0.532 (0.411–0.690)	
≥ 65	0.391 (0.323–0.474)		0.379 (0.285–0.505)	
**Sex**				
male	Reference	< .0001	Reference	< .0001
female	1.475 (1.315–1.654)		1.424 (1.208–1.678)	
**Diabetes mellitus**				
No	Reference	0.0003	Reference	0.5734
Yes	0.807 (0.719–0.906)		0.938 (0.799–1.101)	
**eGFR (ml/min/1.73m**^**2**^**)**				
≥ 60	Reference	0.0419	Reference	0.8034
≥ 45 - < 60	0.726 (0.621–0.849)		0.962 (0.725–1.276)	
≥ 30 - < 45	0.790 (0.677–0.921)		0.882 (0.722–1.078)	
< 30	0.810 (0.649–1.010)		0.791 (0.645–0.969)	
**Albuminuria (mg/g)**				
< 30	Reference	< .0001	Reference	< .0001
≥ 30 - < 300	0.778 (0.680–0.891)		0.735 (0.621–0.871)	
≥ 300	0.632 (0.552–0.725)		0.488 (0.406–0.587)	
**Marital status**				
Married	Reference	0.1975	Reference	0.7603
Single	1.323 (1.089–1.609)		1.122 (0.862–1.459)	
Separated/divorced	1.144 (0.919–1.424)		1.067 (0.808–1.409)	
Widowed	0.938 (0.751–1.172)		0.939 (0.703–1.256)	
**Education level**				
Low	Reference	0.0018	Reference	0.7746
Intermediate	1.200 (1.053–1.367)		1.064 (0.898–1.260)	
High	1.231 (1.053–1.440)		1.011 (0.818–1.249)	
other	1.242 (0.835–1.849)		0.950 (0.571–1.582)	
**Annual gross income**				
< 25.000 €	Reference	< .0001	Reference	0.0134
25.000–50.000 €	1.303 (1.129–1.503)		1.273 (1.082–1.498)	
50.000–100.000 €	1.373 (1.124–1.678)		1.286 (1.011–1.636)	
> 100.000 €	1.653 (1.038–2.632)		1.635 (0.968–2.762)	
**Insurance status**				
Statutory	Reference	0.5781	Reference	0.6973
Private	1.078 (0.865–1.342)		1.081 (0.824–1.418)	
unkown	0.517 (0.047–5.709)		0.616 (0.053–7.116)	
**Body mass index**				
≥18.5 - <25	Reference	0.0050	Reference	0.0716
<18.5	2.490 (0.980–6.327)		2.320 (0.723–7.451)	
≥25 - <30	0.742 (0.632–0.870)		0.851 (0.693–1.045)	
≥30	0.789 (0.676–0.920)		0.859 (0.696–1.060)	
**Smoking status**				
Non smoker	Reference	0.1296	Reference	0.7656
Former smoker	0.913 (0.808–1.030)		1.296 (1.046–1.606)	
Current smoker	1.220 (1.034–1.439)		1.041 (0.888–1.221)	
**Alcohol intake**				
No or very little	Reference	0.0007	Reference	0.9754
Moderate or high	0.781 (0.678–0.900)		1.004 (0.837–1.204)	
**Cardivascular disease**				
No	Reference	0.2792	Reference	0.0042
Yes	0.937 (0.832–1.054)		1.343 (1.093–1.650)	
**Cerebrovascular disease**				
No	Reference	0.2248	Reference	0.5527
Yes	0.891 (0.740–1.073)		0.924 (0.706–1.211)	
**Peripheral vascular disease**				
No	Reference	0.0025	Reference	0.0072
Yes	0.746 (0.616–0.902)		0.695 (0.529–0.912)	
**Antihypertensive Medication**				
** **	Reference*		Reference*	
**RAS inhibitors (ACEI, or ARB or RI)**	1.504 (1.295–1.747)	< .0001	1.617 (1.335–1.960)	< .0001
**Beta blocker**	0.903 (0.807–1.010)	0.0745	0.963 (0.827–1.122)	0.5326
**Ca channel blocker**	0.718 (0.640–0.804)	< .0001	0.892 (0.767–1.038)	0.1374
**Vasodilator**	0.946 (0.721–1.241)	0.6892	0.937 (0.664–1.324)	0.7602
**Central Antiadrenergic**	0.529 (0.445–0.630)	< .0001	0.678 (0.542–0.848)	0.0006
**Peripheral Antiadrenergic**	0.514 (0.406–0.651)	< .0001	0.614 (0.456–0.825)	0.0013
**Diuretics (total)**	1.133 (1.010–1.270)	0.0336	1.460 (1.245–1.712)	< .0001

Data indicate the odds ratio for a successful control of blood pressure among hypertensive patients. Ninety-five % confidence intervals (CI) are shown in brackets. The results of univariate analyses are shown in columns 2 and 3, and the results of the multivariate analysis in columns 4 and 5.

* indicates that for the antihypertensive medications, the Reference population comprised all hypertensive patients not treated with the respective antihypertensive drug class.

**Fig 5:**
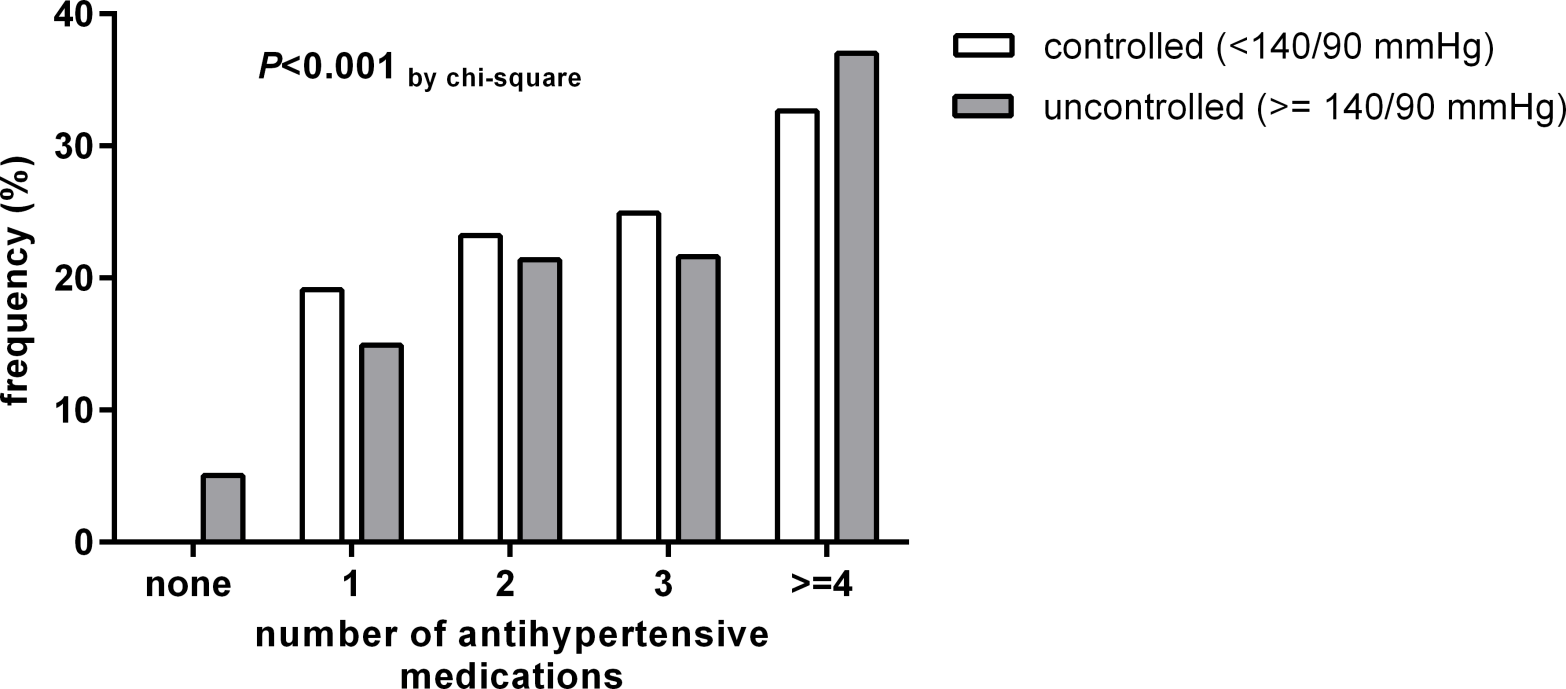
Number of different antihypertensive medications in patients with controlled or uncontrolled hypertension.

Also, we analyzed the medication pattern with regard to the control of blood pressure (Fig 5). Patients with controlled hypertension more often used 1–3 antihypertensive medications than patients with uncontrolled hypertension. In contrast, patients with uncontrolled hypertension more often used 4 or more different antihypertensive medications than patients with controlled hypertension. Of the 2529 patients with uncontrolled hypertension, 1291 (51.0%) met the criteria for resistant hypertension (3 or more different antihypertensive medications including a diuretic). The multivariate logistic regression for control of blood pressure according to the medications used (Table 3) showed that RAS inhibitors and diuretics were associated with a better chance of control whereas alpha blockers and central sympatholytic agents appeared to be associated with a poorer chance of control.

## Discussion

This cross-sectional analysis of a large cohort of CKD patients under nephrology specialist care in Germany confirmed the near universal prevalence of hypertension in CKD patients. Despite very high rates of hypertension awareness and treatment, the latter mostly employing combination pharmacotherapy with several antihypertensive drugs, the control of hypertension was disappointing: Only 49.3% of our patients reached the conservative blood pressure goal of < 140/90 mmHg. Analysis of medication patterns indicated that 51.0% of patients with uncontrolled blood pressure (i.e. one quarter of the study population) met the criteria for resistant hypertension.

The very high prevalence of hypertension in CKD is not unexpected and in agreement with previous reports from similar cohorts of CKD patients in high-income countries, e.g. Japan [19], Spain [16], the U.K. [15], and the U.S. [13]. Although some sociodemographic factors and comorbidities were associated with prevalence rates of uncontrolled hypertension in a statistically significant manner, these effects were generally small, and even the sub-groups with a lower prevalence of hypertension still exhibited a prevalence of uncontrolled hypertension of 90% or more. While unawareness of hypertension is a well-known obstacle for blood pressure control in the general population, rates of hypertension awareness and treatment were high in the present cohort, as reported in other CKD cohorts [13, 16, 20]. This observation renders the relatively low percentage of patients with controlled blood pressure all the more disappointing.

Rates of control of blood pressure in CKD cohorts vary widely. For example, the investigators of the U.S. Chronic Renal Insufficiency Cohort (CRIC) study reported that 67.1% of their patients had blood pressure levels < 140/90 mmHg [13]. In contrast, a large cohort of CKD patients in a different setting in the U.S. described blood pressure control < 140/90 mmHg in only 11–21% of the patients (depending on CKD stage) [20]. In another CKD cohort derived from the U.S. National Health and Nutrition Examination Survey 1996–2006, 48.5% of the participants exhibited controlled blood pressure (defined as blood pressure < 140/90 mmHg) [21]. A cross-sectional survey of CKD patients in Spain found blood pressure levels < 140/90 mmHg in 45.5% of participants [22]. Different inclusion criteria with regard to CKD stages or different settings for recruitment, visits and blood pressure measurements may contribute to the varying control rates.

Within our study higher age and male sex were associated with lower odds for a successful control of hypertension, as also described in other studies of CKD patients [13, 20, 23]. The eGFR stratum (or the CKD stage, conversely) did not affect the odds for control of hypertension after adjustment for other variables, as also reported from the CRIC study [13]. Other authors [20], however, reported conflicting findings. Of particular concern is the lower control rate in patients with higher proteinuria in our and several other reports [13, 22, 23], because these are the very patients who would presumably benefit most from a good control of blood pressure [5]. Somewhat surprising to us was the clear and strong association of higher self-declared yearly gross income with better control of blood pressure. The association by itself was not unexpected, as described e.g. in the CRIC study [13], but the relationship was clearly more marked as in CRIC, despite Germany’s mandatory universal health insurance [24]. Please note that there were a significant number of patients for which we did not have information on annual gross income. We assume that some patients did not feel comfortable to reveal their level of income. All patients had access to nephrology specialist care (GCKD inclusion criterium) and blood pressure medications are provided at no costs or for small copayments. In Germany, the public discussion about differential access to health services has for years revolved around the type of health insurance (statutory insurance for most of the population versus private insurance for ~ 10%, mostly higher-income persons) [24]. In this regard, it appears to be of note that the type of insurance (statutory versus private) did not affect the odds for control of blood pressure but income still did.

As awareness of hypertension and rates of pharmacological treatment were high, we investigated whether a high prevalence of treatment-resistant hypertension in our CKD patients may account for the low overall rates of controlled blood pressure. Our data show that approximately half of our patients with uncontrolled blood pressure had resistant hypertension. We used criteria for resistant hypertension adapted from the definition in the 2013 European Society of Hypertension / European Society of Cardiology hypertension guidelines [11]: blood pressure ≥ 140 mmHg systolic or 90 mmHg diastolic despite at least 3 different antihypertensive medications, including a diuretic. A recent report from the CRIC study used a slightly different definition of “apparent treatment resistant hypertension” and demonstrated that resistant hypertension at enrolment was associated with increased mortality as well as elevated risks for cardiovascular and renal events [25].

An important limitation of our study is that we collected information on the prescribed medications but not on the drug doses, intake schedules, or duration of treatment. Since we also did not assess the adherence to treatment, which is difficult to measure [26], we cannot exclude that the actual drug intake may markedly fall short of the number of drugs prescribed [27]. A previous questionnaire-based estimate of the adherence indicated that patients with CKD exhibited no better adherence regarding antihypertensive medications than hypertensive patients without CKD [28]. Others have reported lower adherence with worsening GFR [29]. On the other hand it is important to note that information on medication intake was directly obtained from patients during standardized interviews rather than from prescription information in health records or from the treating physicians, which may have reduced differences between medication information and actual intake. Nevertheless, our estimate of the frequency of resistant hypertension is likely to be biased by nonadherence [30].

Conversely, our results indicate that 49.0% of the patients with uncontrolled hypertension did not meet the definition of resistant hypertension. While this implies that there was room for use of more anti-hypertensive agents, the overall anti-hypertensive medication use was already substantial. More than 80% of the hypertensive patients received RAS inhibitors, as recommended by many guidelines [10, 11]. The relatively high percentage of patients receiving aliskiren and/or double RAS blockade is probably due to the fact that the data were collected before the publication of the results of the ALTITUDE [31] and VA-NEPHRON-D [32] trials which cautioned against double RAS blockade. The frequency of use of RAS inhibitors as well as of other antihypertensive drug classes was broadly similar to the respective frequencies in CKD cohort studies from Spain [16] and the U.S. [13]. In contrast, a survey-based study of CKD patients identified from the U.S. National Health and Nutrition Examination Survey reported far lower frequencies of RAS blocker usage [33], suggesting that the prescription of these drugs is associated with nephrology care. In our study as well as in CRIC [13], use of RAS inhibitors was associated with better odds for blood pressure control. Several infrequently used drug classes were associated with a lower odds ratio for blood pressure control but this should not be misunderstood to imply a causal relationship. Presumably these drugs were mostly employed as third-line agents in patients with otherwise poorly controlled hypertension. Diuretics were used with similar frequencies in the CRIC cohort [13] and in patients from the GCKD study, and were associated with better control of blood pressure. Again, this observation should not be construed to imply a cause-and-effect relationship; the design of our study does not permit such a conclusion.

A further limitation of our study is the use of office blood pressure measurements obtained during a single visit, even though they were obtained in a highly standardized way. A study which compared office and 24 hour ambulatory blood pressure measurements in CKD patients from a large registry in Spain recorded disagreement between both measurements in a third of the patients [34]. Similar results were recently reported from the CRIC study: only 4.1% of patients had controlled 24-hour ambulatory blood pressure but uncontrolled office blood pressure, i.e. “white-coat” hypertension, whereas no fewer than 27.8% exhibited “masked” hypertension, i.e. uncontrolled ambulatory blood pressure despite controlled office blood pressure [35]. In light of these recent data, we speculate that the control of blood pressure in our patients may be even worse than our office blood pressure results would imply.

In summary, our data clearly show that blood pressure control of CKD patients remains challenging even in the setting of nephrology specialist care, despite high rates of awareness and medication use. The fact that our observations are very similar to those in CRIC, a large, multi-ethnic cohort in the U.S. where patients were enrolled in large centers rather than small practices, indicates that this challenge exists independent of ethnicity, differences in environmental factors, health care coverage and treatment settings. Given that poorly controlled blood pressure is closely associated with progression of kidney disease as well as cardiovascular complications, intensified efforts to control blood pressure appear warranted.
