# Correction: Blood pressure control in chronic kidney disease: A cross-sectional analysis from the German Chronic Kidney Disease (GCKD) study

**DOI:** 10.1371/journal.pone.0204340

**Published:** 2018-09-13

**Authors:** Markus P. Schneider, Karl F. Hilgers, Matthias Schmid, Silvia Hübner, Jennifer Nadal, David Seitz, Martin Busch, Hermann Haller, Anna Köttgen, Florian Kronenberg, Seema Baid-Agrawal, Georg Schlieper, Ulla Schultheiss, Thomas Sitter, Claudia Sommerer, Stephanie Titze, Heike Meiselbach, Christoph Wanner, Kai-Uwe Eckardt, for the GCKD Study Investigators

The affiliation for the nineteenth author is incorrect. Kai-Uwe Eckardt is not affiliated with #7. The correct affiliations are #1 and #8, Department of Nephrology and Department of Nephrology and Hypertension, University Hospital Erlangen, Friedrich-Alexander Universität Erlangen-Nürnberg, Erlangen, Germany and Medical Intensive Care, Charité -Universitätsmedizin Berlin, Berlin, Germany
